# Acute Flaccid Myelitis in Children in Zhejiang Province, China

**DOI:** 10.3389/fneur.2020.00360

**Published:** 2020-05-22

**Authors:** Liming Gong, Yilong Wang, Weiqing Zhang, Chen Chen, Xinghui Yang, Lu Xu, Congying Zhao, Lihua Jiang, Zhefeng Yuan, Zhezhi Xia, Peifang Jiang, Qiong Ge, Juying Yan, Yi Sun, Yin Chen, Zhengyan Zhao, Yanjun Zhang, Feng Gao

**Affiliations:** ^1^Zhejiang Provincial Centre for Disease Control and Prevention, Hangzhou, China; ^2^Department of Neurology, Children's Hospital, Zhejiang University School of Medicine, National Clinical Research Center for Child Health, Hangzhou, China; ^3^Children's Hospital, Zhejiang University School of Medicine, National Clinical Research Center for Child Health, Hangzhou, China; ^4^Department of Radiology, Children's Hospital, Zhejiang University School of Medicine, National Clinical Research Center for Child Health, Hangzhou, China

**Keywords:** acute flaccid myelitis, enterovirus, spinal gray matter, pleocytosis, magnetic resonance imaging, cerebrospinal fluid

## Abstract

In July–December 2018, an outbreak of polio-like acute flaccid myelitis (AFM) occurred in Zhejiang province, China. Enterovirus (EV)-D68 infection has been reported to be associated with AFM. This study aimed to investigate the clinical presentation, laboratory findings, and outcomes of AFM patients. We investigated the clinical and virologic information regarding the AFM patients, and real-time PCR, sequencing, and phylogenetic analysis were used to investigate the cause of AFM. Eighteen cases met the definition of AFM, with a median age of 4.05 years (range, 0.9–9 years), and nine (50%) were EV-D68 positive. Symptoms included acute flaccid limb weakness and cranial nerve dysfunction. On magnetic resonance imaging, 11 (61.1%) patients had spinal gray matter abnormalities. Electromyography results of 16 out of 17 patients (94.1%) were abnormal. Cerebrospinal fluid (CSF) pleocytosis was common (94.4%), while CSF protein concentration was normal in all patients. There was little improvement after early aggressive therapy. Phylogenetic analysis revealed that EV-D68 subclade B3 was the predominant lineage circulating in Zhejiang province in 2018.

## Introduction

The Global Polio Eradication Initiative (GPEI), which was launched in response to a directive from the World Health Assembly, has dramatically reduced the number of cases of wild poliovirus [which is an enterovirus (EV)] globally, including in China (1, 2). The GPEI uses both the oral polio vaccine (which consists of live attenuated poliovirus strains) and the inactivated polio vaccine, both of which are very effective. Based on the high oral polio vaccine coverage, China eradicated polio in October 2000 (3).

Acute flaccid paralysis (AFP), including acute flaccid myelitis (AFM), Guillain–Barré syndrome, acute transverse myelitis, acute disseminated encephalomyelitis (ADEM), toxic neuropathy, and other muscle disorders, is defined as the sudden onset of paralysis or weakness in any part of the body of a child aged <15 years (4). Clusters of AFP have become uncommon, although infection is still a primary cause. Wild poliovirus is no longer the principal cause of AFP; instead, EVs, flaviviruses, herpesviruses, and adenoviruses have become the common etiological factors associated with AFP (5).

AFM is characterized by rapid onset of weakness in one or more limbs and injury to the anterior horn of the spinal cord with or without brainstem involvement (4, 6, 7). Confirmed AFM is defined as acute focal limb weakness and evidence of distinct abnormalities of the spinal cord gray matter on magnetic resonance imaging (MRI) (4). Probable AFM is defined as acute focal limb weakness and cerebrospinal fluid (CSF) pleocytosis (white blood cell count >5 cells/μl) (4).

In 2014, an outbreak of EV-D68 infection, an emerging infectious disease, was determined to be associated with a cluster of AFP cases in children in Colorado, USA (8), with subsequent cases being reported in a number of other countries (5, 9, 10). Several large outbreaks of EV-D68 were associated temporally and geographically with AFM outbreaks (11–14). Application of the Bradford Hill criteria (for establishing causal relationships) indicated that EV-D68 causes AFM (15). Although EV-D68 was not consistently tested for in all patients with confirmed AFM, and when it was, respiratory samples were primarily used (7, 16).

In July–December 2018, an outbreak of cases involving weakness in one or more limbs occurred in our hospital. Hence, we present information on the cluster of AFM cases in our hospital during that time, including the clinical features, laboratory findings, management, and short-term outcomes.

## Patients and Methods

### Patients

Based on this national AFP surveillance system in China (17), we conducted this study in collaboration with clinicians and disease control personnel. An AFM expert panel, consisting of pediatricians and neurologists from CDC and US academic centers, classified patients as confirmed or probable. Confirmed AFM was defined by onset of acute focal limb weakness and evidence of spinal cord lesion with predominant gray matter involvement on MRI. Probable AFM was defined by acute focal limb weakness and a CSF profile showing pleocytosis with leukocyte count >5 cells/μl. Children with new onset of acute focal limb weakness plus evidence of distinct spinal cord gray matter abnormalities on MRI or CSF pleocytosis (white blood cell count >5 cells/μl) were included in the study. Cases of Guillain–Barré syndrome, acute transverse myelitis, and ADEM, and patients who met the clinical case criteria but could not be classified as confirmed or probable cases were excluded from the primary analysis (18–20). Whole blood, CSF, nasopharyngeal swabs, and stool samples were collected, where available. All clinical specimens were stored at −80°C until analysis.

### MRI and Electromyography (EMG) Assessments

In all cases, MRI of the brain and spine (3.0 T) was conducted and the results were reviewed by a senior neurologist and radiologist. EMG and nerve conduction velocity assessments were performed using a Dantec Keypoint® EMG/EP Workstation (with a 3-channel amplifier) and the results were reviewed by an expert in neuroelectrophysiology.

### Standard Protocol Approvals and Patient Consents

Written informed consent for enrolment in this study was obtained from patients and their guardians (children aged >7 years) or the patients' guardians (children aged <7 years). The study was conducted in accordance with the Declaration of Helsinki, and the protocol was approved by the Ethics Committee of the Children's Hospital of Zhejiang University School of Medicine and Zhejiang Provincial Center for Disease Control and Prevention (2019-IRB-054).

### Pathogen Detection

Total DNA/RNA was extracted from 200 μl of clinical specimens using a QIAamp MinElute Virus Spin Kit (Qiagen, Hilden, Germany) according to the manufacturer's protocol. Real-time (RT)-PCR or PCR assay panels (21–23), the FilmArray Respiratory Panel (RP) assay, and the FilmArray Meningitis-Encephalitis (ME) Panel (BioFire Inc., Salt Lake City, UT, USA) were used to detect and assess the titers of the following viruses: human enterovirus (HEV); human rhinovirus (HRV); influenza virus A and B (including subtype determination); human adenovirus; human parainfluenza virus (hPIV) types 1–4; respiratory syncytial virus (RSV) types A and B; human coronavirus (hCoV)-OC43,−229E, -NL63, and -HKU1; human metapneumovirus (hMPV); measles virus; mumps virus; herpes simplex virus; and West Nile virus. Additionally, for EV detection, the extracted samples were amplified and sequenced using RT-PCR, with primers targeting the 5′ untranslated region (UTR) (24). EV-positive samples were subjected to confirmatory EV-D68 assessment by RT-PCR (24), and the VP1 of EV-D68 strains detected from the clinical specimens were amplified and sequenced using the primers and the strategy as described previously (2). The sequences were assembled using Sequencer software version 4.6 (Gene Codes Corporation, Ann Arbor, MI, USA) and aligned with MEGA version 5.

### Phylogenetic Analysis

Phylogenetic analysis was performed using the maximum likelihood (ML) method in MEGA version 5, with bootstrap analysis of 1,000 replicates. A total of 59 EV-D68 strains (31 complete genomes and 28 VP1 sequences) from China, Japan, New Zealand, Italy, and USA, comprising 50 sequences from GenBank and 9 from the clinical samples in this study, were used in the analysis. The EV-D68 sequences originated in this study were deposited in the GenBank database (accession numbers: MK614087).

## Results

**Figure 1:**
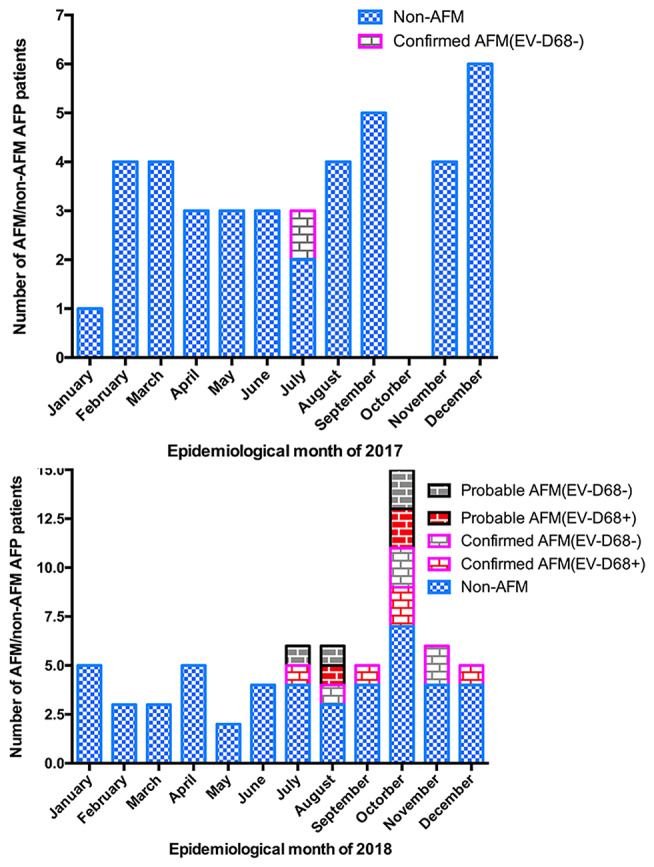
Number of AFM cases by month of limb weakness onset. **(A)** AFM, and AFP cases not meeting the definition of AFM (non-AFM AFP), in January–December 2017. **(B)** AFM, and AFP cases not meeting the definition of AFM (non-AFM AFP), in January–December 2018.

Between January 1, 2017, and December 31, 2018, 107 AFP cases at the Children's Hospital of Zhejiang University School of Medicine were reported to the national AFP surveillance system. There were 42 AFP cases in 2017 and 65 AFP cases in 2018. In total, 18 cases met the definition of AFM (11 confirmed and 7 probable cases), with 1 case in 2017 and 17 in 2018. The others were designated as non-AFM AFP cases, and mostly consisted of Guillain–Barré syndrome, acute transverse myelitis, and ADEM (Figure 1). The clinical features and auxiliary examination results of the 18 AFM patients treated in 2017 and 2018 are summarized in Figure S1.

### Pathogen Detection

Among all cases tested, 9/18 were EV-D68 positive: (7 from stool, 1 from nasopharyngeal swab and stool sample, 1 from oropharyngeal swab and stool samples), while 6/18 were positive for other EVs/rhinoviruses (Table 1). None of the nine EV-D68-positive cases had positive CSF or whole blood specimens (based on either the EV PCR assay or the EV-D68-specific RT-PCR assay with confirmatory VP1 sequencing). In addition, two cases in which EV-D68 was detected in stool samples (based on the EV PCR assay and comparison with EV sequences in GenBank) did not have positive results regarding the confirmatory VP1 sequencing, and the viral titers were much lower in these cases than in the other EV-D68-positive cases. Out of the nine EV-D68-positive samples, only one sample (containing the strain designated “18-128”), which was from a nasopharyngeal swab, was suitable for full genomic analysis (Figure S2). As shown in Figure S3, Fisher's Exact Test analysis revealed no association of EV-D68 detection rate between confirmed AFM patients and probable AFM cases (*p* > 0.05). However, it is really hard to reach a conclusion based on the small sample sizes, only of 18 patients in this study. In addition, our study did not detect EV-D68 in non-AFM patients so far and large numbers of clinical and epidemiological evidence have demonstrated that EV-D68 is a significant cause of AFM (25). By the way, among all cases tested, 4/18 were EV positive but EV-D68 negative and 2/18 were rhinovirus positive in stool samples (Table 1).

**Table 1:** Demographics and clinical statistics of AFM patients (*n* = 18).

**Characteristic**		**No. (%)**
Age at onset (*n* = 18)		
	Median age (range; IQR) (*n* = 18)	4.05(0.9-9)
Sex (*n* = 18)		
	Male	7(38.9)
	Female	11(61.1)
Hospitalized (*n* = 18)		
	Median length of stay	16(5-22)
	Patients with febrile illness preceding limb disorders	16(83.3)
	Patients with respiratory symptoms preceding limb weakness	10(55.6)
	Ventilator support needed	0(0)
	Feeding support needed	0(0)
Limb paralysis (*n* = 18)		
	1 limb	12(66.7)
	2 limbs	4(22.2)
	3 limbs	1(5.5)
	4 limbs	1(5.5)
	Arms only	13(66.7)
	Legs	4(22.2)
AFM cases (*n* = 18)		
	Confirmed cases	11(61.1)
	Probable cases	7(38.9)
Cranial nerve weakness (*n* = 18)		
	Dysphagia	1(5.5)
	Diplopia	0(0)
	Facial disorders	1(5.5)
Neurological symptoms (*n* = 18)		
	Headache	2(10.5)
	Neck stiffness	3(15.7)
	Altered mental status	0(0)
	Seizures during illness	1(5.3)
Treatment (*n* = 18)		
	Experimental antiviral	18(100)
	Systemic corticosteroids	18(100)
	Intravenous immunoglobulin	12(66.7)
Maximum extent of weakness (out of 5 for strength) (*n* = 18)		
	≤ 2/5 strength	14(77.8)
	3/5 strength	2(11.1)
	>3/5 strength	3(16.7)
Clinical outcome (*n* = 18)		
	As weak	10(55.6)
	Some improvement	7(38.8)
	Full recovery	0(0)
	Weaker	1(5.5)
Virology (EV-D68 positive)		9(50.0)
	Stool	8(44.4)
	Blood	0(0)
	CSF	0(0)
	NP/OP	2(11.1)
Virology (enterovirus positive EV-D68 negative)	Stool	4(22.2)
Virology (rhinovirus positive)	Stool	2(11.1)

*IQR, interquartile range; CSF, cerebrospinal fluid; NP, nasopharyngeal swab; OP, oropharyngeal swab*.

### Phylogenetic and Sequence Analysis

**Figure 2:**
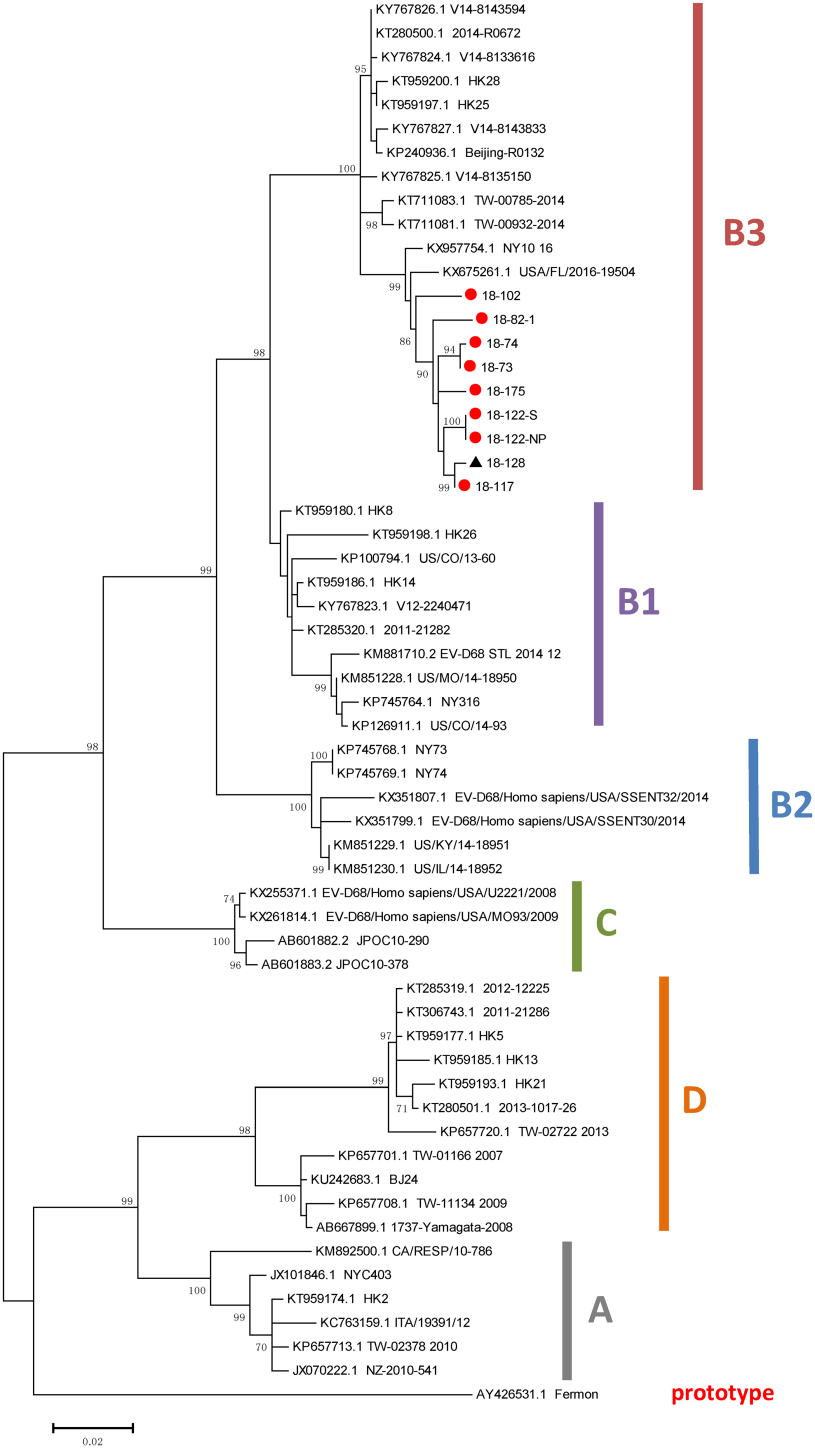
Phylogenetic analysis of VP1 sequences of EV-D68 strains. The trees were constructed based on VP1 sequences using the ML method, with bootstrap values calculated from 1,000 trees. Red circles indicate EV-D68 strains obtained in the present study. EV, enterovirus; ML, maximum likelihood.

A phylogenetic analysis of the VP1 region of EV-D68 genomes was conducted, which involved 59 VP1 sequences, including nine obtained from the EV-D68-positive patients in this study. The results suggested that all nine strains detected in this study belonged to subclade B3 (Figure 2). The nine VP1 sequences detected in this study had nucleotide identities with each other of 98–100%; the VP1 sequence of the strain designated “18-128” had the lowest nucleotide identity (98%).

### Clinical Characteristics of AFM

The clinical features of the AFM patients are summarized in Table 1. All the patients were children hospitalized due to neurological symptoms. There were 7 males and 11 females, with a median age of 4.05 years (range, 0.9–9 years).

Regarding the symptoms before onset of AFM, 15/18 patients had fever and 10/18 experienced a prodromal respiratory symptom. Regarding the other symptoms, 4/18 patients had pain in the affected limbs, 2 (11.1%) had intact sensation in the affected limbs, 2/18 had headache, 1/18 had seizures, and none had altered mental state, which demonstrated that cerebral involvement is rare in AFM. No patients needed mechanical ventilation; no patients had signs of sphincter dysfunction.

Regarding the limbs involved, 12/18 patients had weakness in the arms (two of which also had cranial nerve dysfunction), 4/18 had weakness in only the legs, and 2/18 had weakness in both the arms and legs. In 15 patients, the degree of limb weakness (on a 5-point scale, where 0 represents complete paralysis and no muscle contraction and 5 represents muscle strength is normal) ranged from 0 to 3, and only three patients had a score >3. In our study, AFM patients also showed weakening or disappearance of deep reflexes and muscle atrophy.

### MRI and EMG Results

**Figure 3:**
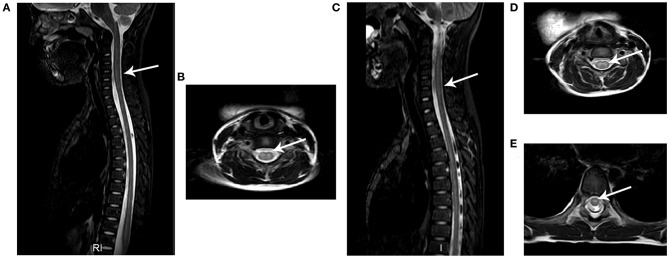
Typical MRI results of AFM patients. The images from two AFM patients (case a: **A,B**; case b: **C,E**) after onset of neurological symptoms. **(A,C)** Sagittal T2-weighted images showing longitudinal hyperintensity (arrow) in central gray matter. **(B,D,E)** Axial T2-weighted sequences showing hyperintensity in spinal cord gray matter.

Spinal MRI was performed for all 18 patients and 17 patients underwent brain MRI within 7 days after admission. Consistent with previous reports (13), hyperintensity on T2-weighted images was observed in spinal gray matter in confirmed AFM cases. As demonstrated in Figure 3 and Table 2, 11/18 patients had spinal gray matter abnormalities, mainly with anterior horn involvement. Longitudinal cord lesions spanning a median of 4 vertebral levels (range, 2–13) were observed in all confirmed AFM cases, and the cervical spine was the most often affected region (9/11). Brainstem lesions were found in three (17.6%) cases involving focal lesions in the pons (*n* = 1), medulla oblongata (*n* = 2), and one (5.9%) had abnormalities in cortical gray matter (*n* = 1). Five of 11 confirmed AFM cases were EV-D68 positive, the length of spinal lesion of EV-D68-positive patients was spanning a median of 3 spinal segments (range, 2–5 spinal segments), and EV-D68 negative patients were spanning a median of 7 spinal segments (range, 3–13 spinal segments); however, there is no difference of spinal lesion between EV-D68-positive cases and -negative cases (*p* > 0.05).

**Table 2:** Results of MRI, EMG, CSF statistics of AFM patients.

**Characteristic**		**No. (%)**
MRI		
Lesion on spine MRI (*n* = 18)		
	Lesions on spinal cord MRI	11 (61.1)
	Cervical spinal cord involvement^**a**^	9 (81.1)
	Thoracic cord involvement^**a**^	6 (36.3)
	Lumbar spinal cord involvement^**a**^	1 (9.1)
	Sacral cord involvement^**a**^	0
	Median length of spinal lesion, No. of vertebral levels (IQR)	4 (2-13)
	Median length of spinal lesion, No. of vertebral levels (IQR), EV-D68 positive	3 (2-5)
	Median length of spinal lesion, No. of vertebral levels (IQR), EV-D68 negative	7 (3-13)
Lesions on brain MRI (*n* = 17)		
	Cortical gray matter	1 (5.9)
	Subcortical white matter	3 (17.6)
	Basal ganglia	0 (0)
	Cerebellum	0 (0)
	Medulla oblongata	2 (11.8)
	Midbrian	0 (0)
	Pons	1 (5.9)
EMG (*n* = 17)		
Motor nerve conduction study,		
	Diminished motor conduction velocity	4 (23.5)
	Absent compound muscle action potential	2 (11.8)
	Diminished compound muscle action potential	9 (52.9)
	Self-generated muscle action potential	14 (82.3)
	Abnormal waveform	10 (58.9)
Sensory nerve conduction study,		
	Absent sensory conduction velocity	0 (0)
	Diminished sensory conduction velocity	0 (0)
F-wave study, no. /total no. of cases (%)		
	Decreased persistence	0 (0)
CSF findings (*n* = 18)		
	Pleocytosis (WBC count >5 cells/μL)	17(94.4)
	high CSF protein concentration (<0.45 g/L)	0(0)
	low glucose concentration(<2.78 nmol/L)	0(0)
	CSF protein concentration(<0.45 g/L) (IQR)	0.22 (0.13-0.44)
	CSF glucose (2.78–4.5 nmol/L) (IQR)	3.99 (2.95-6.41)
	Medial CSF detection number of days(IQR)	3 (1-8)

Data are presented as no. (%) unless otherwise indicated.

CSF, cerebrospinal fluid; IgG, immunoglobulin G; IQR, interquartile range.

MRI, magnetic resonance imaging; WBC, white blood cell.

a *Proportion of confirmed AFM cases*.

EMG and nerve conduction velocity assessments were conducted in 17/18 patients at a median of 10 days (range, 4–15 days) after onset of neurological symptoms. In 17/18 patients at a median of 10 days (range, 4–15 days) after onset of neurological symptoms. Of the 17 patients, 9 had diminished compound muscle action potential, 14 had self-generated muscle action potential, 4 had prolonged nerve conduction velocity accompanied by self-generated muscle action potential and diminished compound muscle action potential in the acute phase, and none had sensory nerve conduction abnormalities or abnormal F-waves. Thus, unlike in Guillain–Barré syndrome, there were no sensory EMG abnormalities in any of the AFM patients (Table 2). Four patients with prolonged nerve conduction velocity quickly returned to normal with EMG assessments at the later follow-up but showed signs of anterior horn cell damage.

### CSF Results

All 18 AFM patients underwent CSF assessment, at a median of 3 days (range, 1–8 days) after the onset of neurological symptoms. Of the 18 patients, 17 had pleocytosis (>5 white blood cells/μl), with a median of 27 cells/μl (range, 5–160 cells/μl). All 18 AFM patients had normal CSF protein concentrations (normal reference range: <0.45 g/L), with a median concentration of 0.22 g/L (range, 0.13–0.44 g/L); only three patients had CSF glucose concentrations >4.5 mmol/L (normal reference range: 2.78–4.5 mmol/L) (Table 2).

### Treatment and Outcomes

All the patients received antiviral therapy (acyclovir or ribavirin) and intravenous methylprednisolone at a dose of 20 mg/kg/day (1 g maximum) for 3–6 days. Additionally, 12 patients received intravenous immunoglobulin at a dose of 1–2 g/kg. The median length of hospital stay was 16 days. There was no obvious improvement after treatment in most patients. Six of nine EV-D68-positive AFM patients and 5/9 EV-D68-negative AFM patients showed no improvement after a series treatment; 3/9 EV-D68-positive AFM patients and 4/9 EV-D68-negative AFM patients had some improvement. The median follow-up period was 17.5 months (interquartile range, 15.8–31.7 months).

## Discussion

AFM, a polio-like subtype of AFP caused by injury to the anterior horn of the spinal cord, has been reported by the USA's CDC to be a consequence of EV-D68 outbreaks, such as the outbreaks in the USA and Canada in 2014, in which the AFM cases mostly involved children (8). At least 14 countries (the USA, Canada, Germany, Denmark, Italy, France, the Netherlands, India, Norway, the UK, Spain, Vietnam, Sweden, and Japan) have reported AFM outbreaks (5, 6, 8–12, 14, 15, 26–30). There have been no reports of AFM in mainland China until now.

At our hospital, there were 17 AFM cases (10 confirmed and 7 probable cases) in 2018, mostly occurred in July–December, and particularly in October. Nine of eighteen AFM patients were EV-D68 positive (seven based on stool samples and two based on nasopharyngeal swabs plus stool samples). The EV-D68-positive rate in our AFM patients was higher than the rates reported in other studies (11, 31). It is possible that the cases mostly occurred in the summer and early autumn, which are the periods associated with an active EV-D68 circulation pattern (32). All the EV-D68 strains identified in this study belonged to clade B3. This is in line with previous findings regarding the EV-D68 epidemics in Hong Kong (2014) (2) and Taiwan (2014) (33), which indicated that EV-D68 subclade B3 was the predominant lineage circulating in China during the epidemics.

Among the AFM cases, 7 children were male and 11 were female. The median age was 4.05 years, which is in line with the results of a national survey of AFM in Japan in 2015 (13). As demonstrated in our study, the symptoms included prodromal respiratory symptoms and neurological manifestations with asymmetric flaccid limb weakness, sometimes accompanied by pain and cranial nerve dysfunction. Before onset of AFM, most patients experienced fever and prodromal respiratory symptoms, which is consistent with the findings of previous studies (10, 12, 13). In the present study, 66.7% of AFM patients had only one limb involved and none had sensory nerve conduction abnormalities. Limb pain was observed in 40–69% of AFM cases in North America during the 2014 outbreak (4), while 10.5% of children in our study had limb pain. Clinical manifestations may help to distinguish AFM from Guillain–Barré syndrome, acute transverse myelitis, and ADEM, but diagnosis based only on clinical manifestations is difficult, especially in young children who do not cooperate with physical examinations.

The definition of AFM is acute focal limb weakness plus evidence of spinal cord lesions on MRI, or CSF pleocytosis (white blood cell count >5 cells/μl). As this was our first encounter with AFM patients, we lacked experience with AFM-related MRI inspection and analyses. We identified 11 patients (61.1%) with lesions on spinal MRI, while in a national AFM survey in 2014 in the USA, 96% of patients had spinal cord involvement (with >1 spinal segment involved) (16). In our study, the cervical spinal cord was involved in nine confirmed AFM cases, the thoracic cord was involved in four, and the lumbar cord was involved in one. A previous AFM study reported mild to moderate CSF pleocytosis with lymphocytic predominance, mildly elevated protein concentration, and normal glucose concentration in most patients (11). In our study, 17 of the 18 patients had mild CSF pleocytosis, which is consistent with the previous research, but all patients had a normal protein concentration and most had a normal glucose concentration, which is slightly different compared to the previous research. Differences in testing standards between countries may be the main reason.

Although EMG results are not included in the AFM definition, our opinion is that the EMG results have great clinical significance regarding AFM diagnoses. In our study, the EMG results were abnormal in 16 of 17 patients, diminished compound muscle action potential was confirmed in 9 patients, self-generated muscle action potential was found in 14 patients, most patients had normal conduction velocity, and no patients had signs of sensory nerve conduction abnormalities; these findings indicated axon damage, mainly with anterior horn involvement. Four patients with prolonged nerve conduction velocity in acute state quick returned to normal with EMG assessments at the later follow-up but still with anterior horn cell damage.

In previous studies (8), AFM patients did not have early clinical responses to intravenous immunoglobulin, high-dose intravenous corticosteroids, or antiviral therapy, and longer-term outcomes have not yet been reported. In our study, high-dose intravenous corticosteroids and antiviral therapy were used for treatment in all the AFM patients and the majority also received immunoglobulin therapy. It is regrettable that there was no obvious improvement in many patients after these treatments were administered.

Recently, Zhang et al. (34) established a mouse model of EV-D68 infection using Institute of Cancer Research (ICR) suckling mice, and they found that EV-D68 strains cause paralytic myelitis and the spinal cord is the major site of viral replication. These observations match a previous finding that EV-D68 can infect and kill motor neurons in the spinal cord of neonatal mice (35, 36).

We did not detect EV-D68 in the CSF or whole blood specimens from the nine EV-D68-positive patients. Previous studies have determined that CSF detection rates for known neurotropic EVs (such as polioviruses and EV-A71) are as low as 0–5% (37, 38). In addition, the delayed collection of clinical specimens relative to symptom onset (2–19 days) may have led to actual low viral loads or RNA degradation, thus resulting in insufficient EV-D68 RNA levels for detection (Figure S1).

## Conclusion

Infections with EVs have occurred more frequently in recent years, and they have been associated with severe clinical courses (12–14). Central nervous system infections involving the non-polio EV, EV-D68, have been regarded as the main reason for EV-associated AFP in recent years (15), but these infections have no effective treatment in the acute stage. In our research, 18 cases met the definition of AFM (11 confirmed and 7 probable cases), 12/18 patients had weakness in the arms, 4/18 had weakness in only the legs, and 2/18 had weakness in both the arms and legs. Nine of eighteen AFM patients were EV-D68 positive, and there was no obvious improvement after treatment in most AFM patients. Thus, it is important to recognize that AFM and studies are required to develop both novel preventive and therapeutic measures. We believe that a more effective global surveillance is an essential strategy for controlling AFM, and it needs the cooperation of medical workers from different countries.

## Data Availability Statement

All datasets generated for this study are included in the article/Supplementary Material.

## Ethics Statement

The studies involving human participants were reviewed and approved by Ethics Committee of the Children's Hospital of Zhejiang University School of Medicine and Zhejiang Provincial Center for Disease Control and Prevention. Written informed consent to participate in this study was provided by the participants' legal guardian/next of kin.

## Author Contributions

FG and YZ designed the data collection instruments, collected data, carried out the initial analyses, and reviewed and revised the manuscript. YW, LG, WZ, CC, XY, LX, CZ, LJ, ZY, ZX, PJ, QG, JY, YS, YC, and ZZ conceptualized and designed the study, coordinated and supervised data collection, and critically reviewed the manuscript for important intellectual content. All authors approved the final manuscript as submitted and agreed to be accountable for all aspects of the work.

## Conflict of Interest

The authors declare that the research was conducted in the absence of any commercial or financial relationships that could be construed as a potential conflict of interest.

## Supplementary Material

Figure S1 Clinical features and laboratory results.

Figure S2 Detection of enterovirus D68 from different biological samples.

Figure S3 Correlation of AFM with EV-D68 detection rate.
