# Supplementary figures and images for: Acute Flaccid Myelitis in Children in Zhejiang Province, China
Source: Front Neurol. 2020 May 22;11:360. doi: 10.3389/fneur.2020.00360 (PMC7256184; doi:10.3389/fneur.2020.00360)

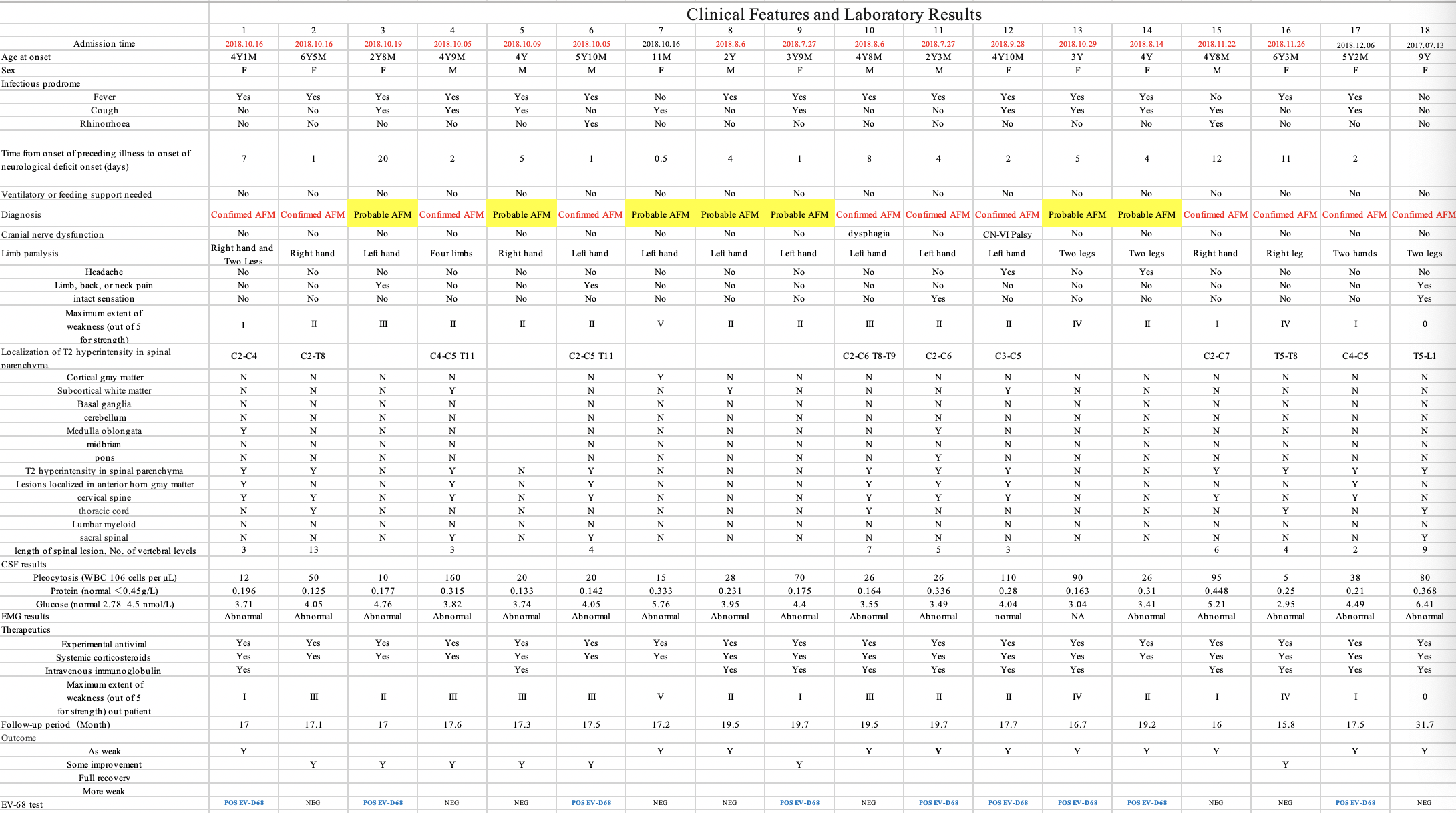

Supplement: Figure S1 — Clinical features and laboratory results. [file Image_1.PNG]

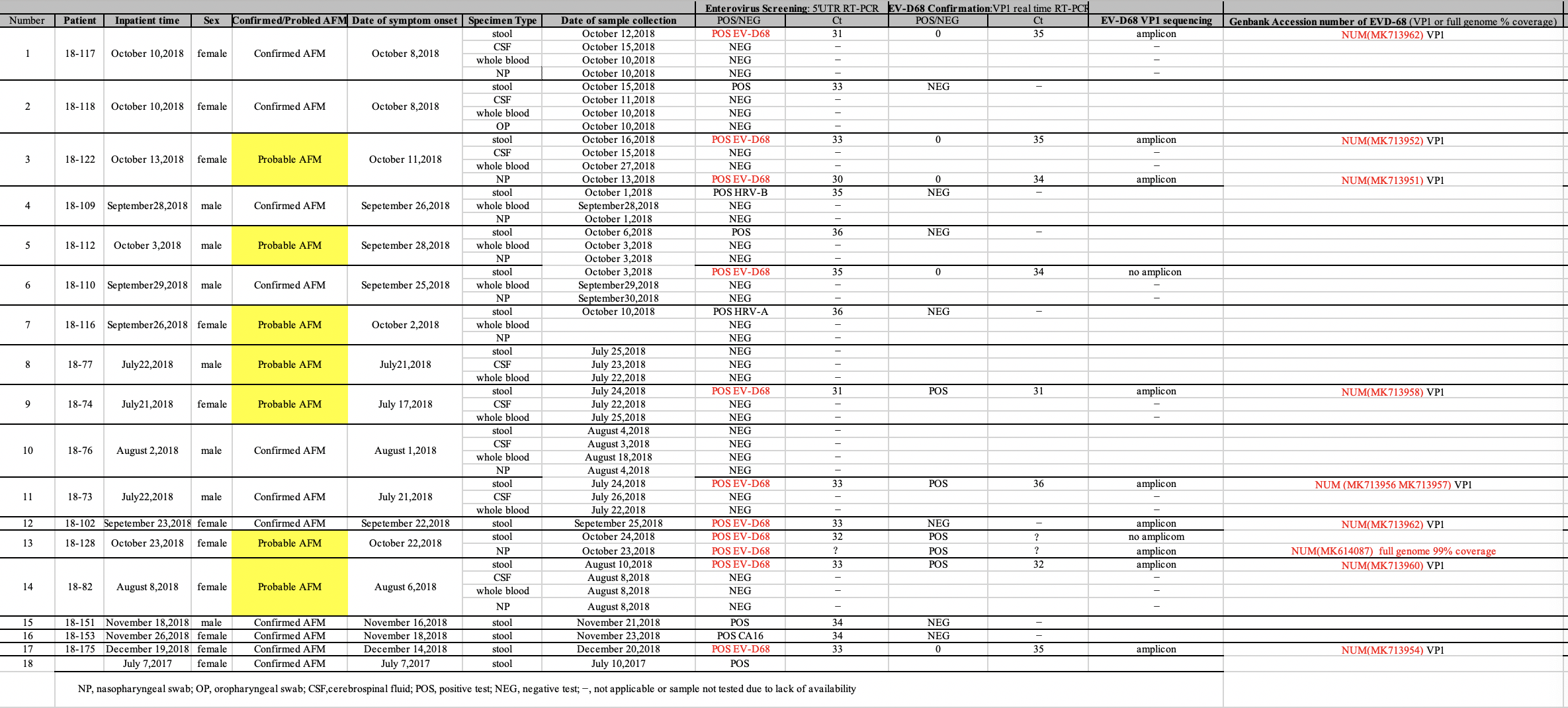

Supplement: Figure S2 — Detection of enterovirus D68 from different biological samples. [file Image_2.PNG]

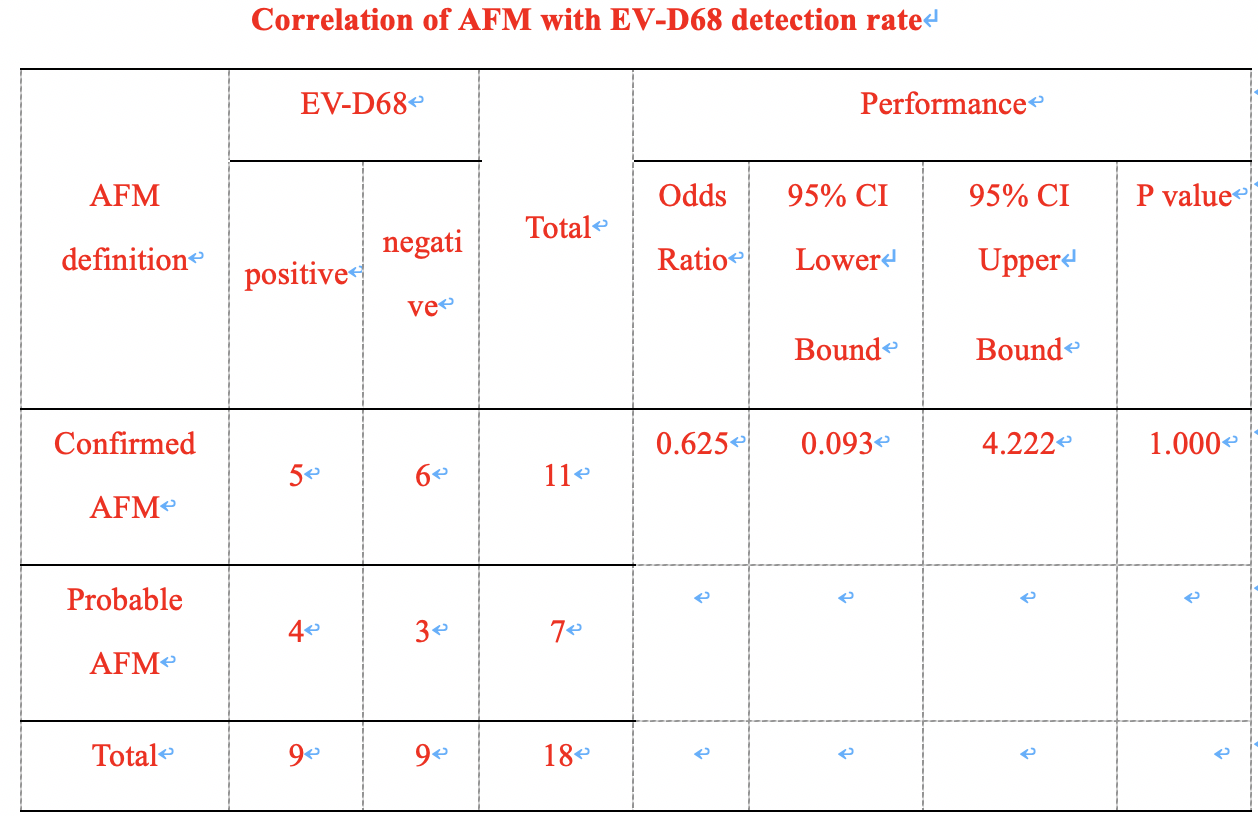

Supplement: Figure S3 — Correlation of AFM with EV-D68 detection rate. [file Image_3.PNG]
