# Supplementary material for: Acute Flaccid Myelitis in Children in Zhejiang Province, China
Source: Front Neurol. 2020 May 22;11:360. doi: 10.3389/fneur.2020.00360 (PMC7256184; doi:10.3389/fneur.2020.00360)
Supplement: Supplementary file 4 [file Table_1.DOCX]

**Table 1.** Demographics and Clinical statistics of AFM patients(n=18)

| Characteristic |  | No. (%) |
| --- | --- | --- |
| Age at onset (n=18) |  |  |
|  | median age (range; IQR) (n=18) | 4.05(0.9-9) |
| Sex (n=18) |  |  |
|  | Male | 7(38.9) |
|  | Female | 11(61.1) |
| Hospitalized (n=18) |  |  |
|  | Median length of stay | 16(5-22) |
|  | Patients with febrile illness preceding limb disorders | 16(83.3) |
|  | Patients with respiratory symptoms preceding limb weakness | 10(55.6) |
|  | Ventilator support needed | 0(0) |
|  | Feeding support needed | 0(0) |
| Limb paralysis (n=18) |  |  |
|  | 1 limb | 12(66.7) |
|  | 2 limbs | 4(22.2) |
|  | 3 limbs | 1(5.5) |
|  | 4 limbs | 1(5.5) |
|  | Arms only | 13(66.7) |
|  | Legs | 4(22.2) |
| AFM cases (n=18) |  |  |
|  | Confirmed cases | 11(61.1) |
|  | Probable cases | 7(38.9) |
| Cranial nerve weakness  (n =18) |  |  |
|  | Dysphagia | 1(5.5) |
|  | Diplopia | 0(0) |
|  | Facial disorders | 1(5.5) |
| Neurological symptoms  (n =18) |  |  |
|  | Headache | 2(10.5) |
|  | Neck stiffness | 3(15.7) |
|  | Altered mental status | 0(0) |
|  | Seizures during illness | 1(5.3) |
| Treatment |  |  |
| (n=18) | Experimental antiviral | 18(100) |
|  | Systemic corticosteroids | 18(100) |
|  | Intravenous immunoglobulin | 12(66.7) |
| Maximum extent of weakness (out of 5 for strength) (n=18) |  |  |
|  | ≤ 2/5 strength | 14(77.8) |
|  | 3/5 strength | 2(11.1) |
|  | ＞3/5 strength | 3(16.7) |
| Clinical outcome (n = 18) |  |  |
|  | As weak | 10(55.6) |
|  | Some improvement | 7(38.8) |
|  | Full recovery | 0(0) |
|  | Weaker | 1(5.5) |
| Virology (EV-D68 positive)  Virology (enterovirus positive EV-D68 negative)  Virology (rhinovirus positive) | Stool  Blood  CSF  NP/OP  Stool  Stool | 9(50.0)  8(44.4)  0(0)  0(0)  2(11.1)  4(22.2)  2(11.1) |

Abbreviation: IQR, interquartile range; CSF, cerebrospinal fluid; NP, nasopharyngeal swab; OP, oropharyngeal swab
