# Supplementary material for: Acute Flaccid Myelitis in Children in Zhejiang Province, China
Source: Front Neurol. 2020 May 22;11:360. doi: 10.3389/fneur.2020.00360 (PMC7256184; doi:10.3389/fneur.2020.00360)
Supplement: Supplementary file 5 [file Table_2.DOCX]

**Table 2.** Results of MRI, EMG, CSF statistics of AFM patients

| Characteristic |  | No. (%) |
| --- | --- | --- |
| MRI |  |  |
| Lesion on spine MRI (n=18) |  |  |
|  | Lesions on spinal cord MRI | 11 (61.1) |
|  | Cervical spinal cord involvement**^a^** | 9 (81.1) |
|  | Thoracic cord involvement**^a^** | 6 (36.3) |
|  | Lumbar spinal cord involvement**^a^** | 1 (9.1) |
|  | Sacral cord involvement**^a^** | 0 |
|  | Median length of spinal lesion, No. of vertebral levels (IQR) | 4 (2-13) |
|  | Median length of spinal lesion, No. of vertebral levels (IQR), EV-D68 positive | 3 (2-5) |
|  | Median length of spinal lesion, No. of vertebral levels (IQR), EV-D68 negative | 7 (3-13) |
| Lesions on brain MRI(n=17) |  |  |
|  | Cortical gray matter | 1 (5.9) |
|  | Subcortical white matter | 3 (17.6) |
|  | Basal ganglia | 0 (0) |
|  | cerebellum | 0 (0) |
|  | Medulla oblongata | 2 (11.8) |
|  | midbrian | 0 (0) |
|  | pons | 1 (5.9) |
| EMG(n=17) |  |  |
| Motor nerve conduction study, |  |  |
|  | Diminished motor conduction velocity | 4 (23.5) |
|  | Absent compound muscle action potential | 2 (11.8) |
|  | Diminished compound muscle action potential | 9 (52.9) |
|  | Self-generated muscle action potential | 14 (82.3) |
|  | Abnormal waveform | 10 (58.9) |
| Sensory nerve conduction study, |  |  |
|  | Absent sensory conduction velocity | 0 (0) |
|  | Diminished sensory conduction velocity | 0 (0) |
| F-wave study, no./total no. of cases (%) |  |  |
|  | Decreased persistence | 0 (0) |
| CSF findings(n=18) |  |  |
|  | Pleocytosis (WBC count >5 cells/μL) | 17(94.4) |
|  | high CSF protein concentration (<0.45g/L) | 0(0) |
|  | low glucose concentration(<2.78nmol/L) | 0(0) |
|  | CSF protein concentration(<0.45g/L) (IQR) | 0.22 (0.13-0.44) |
|  | CSF glucose (2.78–4.5 nmol/L) (IQR) | 3.99 (2.95-6.41) |
|  | Medial CSF detection number of days(IQR) | 3 (1-8) |

Data are presented as no. (%) unless otherwise indicated.

Abbreviations: CSF, cerebrospinal fluid; IgG, immunoglobulin G; IQR, interquartile range;

MRI, magnetic resonance imaging; WBC, white blood cell.

**^a^:** Proportion of confirmed AFM cases
